# Enhancing protein-vitamin binding residues prediction by multiple heterogeneous subspace SVMs ensemble

**DOI:** 10.1186/1471-2105-15-297

**Published:** 2014-09-05

**Authors:** Dong-Jun Yu, Jun Hu, Hui Yan, Xi-Bei Yang, Jing-Yu Yang, Hong-Bin Shen

**Affiliations:** School of Computer Science and Engineering, Nanjing University of Science and Technology, Xiaolingwei 200, Nanjing, 210094 China; Institute of Image Processing and Pattern Recognition, Shanghai Jiao Tong University, Dongchuan Road 800, Shanghai, 200240 China

**Keywords:** Protein-vitamin binding residue, Feature subspace, Heterogeneous SVM, Classifier ensemble

## Abstract

**Background:**

Vitamins are typical ligands that play critical roles in various metabolic processes. The accurate identification of the vitamin-binding residues solely based on a protein sequence is of significant importance for the functional annotation of proteins, especially in the post-genomic era, when large volumes of protein sequences are accumulating quickly without being functionally annotated.

**Results:**

In this paper, a new predictor called TargetVita is designed and implemented for predicting protein-vitamin binding residues using protein sequences. In TargetVita, features derived from the position-specific scoring matrix (PSSM), predicted protein secondary structure, and vitamin binding propensity are combined to form the original feature space; then, several feature subspaces are selected by performing different feature selection methods. Finally, based on the selected feature subspaces, heterogeneous SVMs are trained and then ensembled for performing prediction.

**Conclusions:**

The experimental results obtained with four separate vitamin-binding benchmark datasets demonstrate that the proposed TargetVita is superior to the state-of-the-art vitamin-specific predictor, and an average improvement of 10% in terms of the Matthews correlation coefficient (MCC) was achieved over independent validation tests. The TargetVita web server and the datasets used are freely available for academic use at http://csbio.njust.edu.cn/bioinf/TargetVita or http://www.csbio.sjtu.edu.cn/bioinf/TargetVita.

**Electronic supplementary material:**

The online version of this article (doi:10.1186/1471-2105-15-297) contains supplementary material, which is available to authorized users.

## Background

Functional positions in a protein are residues that play more critical roles than other residues and enable the protein to perform specific biological functions, such as capturing drugs [1], binding ligands [2], and interacting with other proteins [3]. However, functionally annotated proteins still account for only a small portion of sequenced proteins, and the gap between annotated and sequenced proteins is ever-increasing with the rapid development of advanced sequencing technology and concerted genome projects [4]. Automated computational methods for the prediction of protein functional positions are urgently needed and have become a hotspot in bioinformatics research. During the past decades, machine-learning-based computational methods have been extensively applied to various protein functional position prediction problems [5–7].

Protein-ligand interaction is one of the most important protein functions and plays vital roles in virtually all biological processes [2, 8, 9]. Considerable effort has been made to design effective methods for protein-ligand binding residue (site) prediction, and much progress has been made in this area [10]. In the early stage, *general-purpose* protein-ligand predictors, which predict ligand binding sites (pockets) regardless of ligand types, dominate in the fields of protein-ligand binding site prediction. Such predictors include LIGSITE [11], CASTp [12], SURFNET [13], POCKET [14], fpocket [15], Q-SiteFinder [16], and SITEHOUND [17]. Later, researchers observed that protein-ligand binding sites (pockets) vary significantly in their roles, sizes, and distributions for different types of protein-ligand interactions, and different ligands tend to bind diverse types of residues with prominent specificities [18, 19]. These observations motivated the emergence of *ligand-specific* predictors, which are specifically designed to predict binding residues or sites for certain ligand types, such as NsitePred [20] and TargetS [21] for protein-nucleotide binding prediction, FINDSITE-metal [22] and CHED [23] for protein-metal binding prediction, MetaDBSite [24] and DNABR [25] for protein-DNA binding prediction, protein-drug binding prediction [26], and others. These studies have shown that *ligand-specific* predictors are often superior to *general-purpose* predictors and are a promising route for improving the performance of protein-ligand prediction [20, 21].

Vitamins are typical ligands and play critical roles in various metabolic processes [27–29]. However, to the best of our knowledge, minimal work has been performed to design a specific predictor for predicting protein-vitamin binding residues. Recently, Panwar et al. [30] published their pioneering work on protein-vitamin binding prediction and a predictor, called VitaPred, was implemented. VitaPred [30] is a sequence-based *ligand-specific* predictor specifically designed for predicting protein-vitamin binding residues, and it consists of four independent prediction modules for predicting vitamin, vitamin-A, vitamin-B, and pyridoxal-5-phosphate (vitamin-B6) binding residues. VitaPred encodes each residue into a 340-D feature vector by applying a sliding window to the position-specific scoring matrix (PSSM) of a protein sequence; then, a support vector machine (SVM) is trained on the set of feature vectors of all the training residues. In the prediction stage, the feature vector of each residue in a query sequence is fed into the trained SVM, and the binding propensity of each residue is obtained; finally, a threshold is used to determine whether a residue is vitamin-interacting. Although VitaPred achieved great success in predicting protein-vitamin binding residues, there is still room for further improving the prediction performance: first, only the PSSM-derived feature was used in VitaPred, and other valuable features (e.g., protein secondary structure) were not well considered; second, the PSSM feature constructed in VitaPred may contain redundant information, which is useless or even harmful for performing prediction.

This paper follows the pioneering work of Panwar et al. [30] and aims to further improve the performance of protein-vitamin binding residue prediction. A new predictor, called TargetVita, which utilises multiple sequence-derived features and heterogeneous SVMs ensemble based on feature selection, is developed. In TargetVita, three different types of features (i.e., a position-specific scoring matrix feature, a predicted secondary structure feature, and a vitamin binding propensity feature) are combined to form the original feature space; then, three feature selection methods are performed on the original feature space to extract three different feature subspaces, and heterogeneous SVMs are trained on the reduced feature subspaces. Finally, when performing prediction, the vitamin-binding propensity of each residue in a query sequence is predicted by averaging the outputs of the three trained heterogeneous SVMs. Experimental results obtained with four separate vitamin-binding benchmark datasets demonstrate that the proposed TargetVita is superior to VitaPred [30] and an average improvement of 10% in terms of the Matthews correlation coefficient (*MCC*) was achieved over independent validation tests.

## Methods

### Benchmark datasets

In this study, four benchmark datasets created by Panwar et al. [30] were utilised to evaluate the efficacy of the proposed method. For convenience, the four benchmark datasets are denoted as DVI (dataset of vitamin-interacting proteins), DVAI (dataset of vitamin-A-interacting proteins), DVBI (dataset of vitamin-B-interacting proteins), and DPLPI (dataset of pyridoxal-5-phosphate interacting proteins), respectively.

Each benchmark dataset was constructed with a stringent procedure as follows [30]: taking DVI as an example, 1061 PDB IDs of proteins that make contact with vitamins were first collected from SuperSite documentation [31]; then, the sequences of all chains of these 1061 PDB IDs were downloaded from the Protein Data Bank [32]. Among the obtained sequences, 2720 sequences were finally chosen according to the results returned from the Ligand Protein Contact (LPC) web server [33] by taking 1061 PDB IDs as inputs. Then, a threshold of 5.0 Å was used to determine the vitamin-interacting residues: a residue was considered to be vitamin-interacting if the closest distance between atoms of the protein and the partner vitamin was within the threshold (5.0 Å) [30]; finally, the maximal pairwise sequence identity of the vitamin-binding sequences obtained in the above steps was further reduced to 25% by using BLASTCLUST [34], and the obtained 187 vitamin-interacting chains with 30156 vitamin-binding residues constituted the DVI. Similarly, DVAI, DVBI, and DPLPI, which consist of 31, 141, and 71 non-redundant sequences, respectively, were also constructed by repeating the above-mentioned steps.

Four different independent validation datasets for DVI, DVAI, DVBI, and DPLPI, which consist of 46, 15, 27, and 16 non-redundant sequences, respectively, were also constructed [30]. In addition, to guarantee the independence of the independent validation subset, the maximal pairwise sequence identity of each independent validation dataset is less than 25%, and any sequence in an independent validation dataset shares <25% identity to the sequences in the corresponding training dataset. Table 1 summarises the detailed compositions of the four benchmark datasets. Details for constructing these datasets can be found in [30].

**Table 1:** **Compositions of the training datasets and the corresponding independent validation datasets for the 4 types of vitamin-interacting benchmark datasets**

Dataset	Training Dataset		Independent Validation Dataset		Total No. of Sequences
	No. of Sequences	(numP, numN) ^*^	No. of Sequences	(numP, numN) ^*^	
DVI	187	(3016, 62122)	46	(654, 11676)	233
DVAI	31	(538, 7376)	15	(181, 1441)	46
DVBI	141	(2219, 50179)	27	(419, 8947)	168
DPLPI	71	(1092, 26638)	16	(246, 5935)	87

^*^numP and numN represent the numbers of positive (binding) and negative (non-binding) samples, respectively.

To evaluate the performance of TargetVita with non-vitamin binding proteins, we also constructed a non-vitamin binding dataset, denoted as NVD, from BioLip [35], which is the most recently released semi-manually curated database for biologically relevant ligand–protein interactions. We constructed the NVD as follows: First, all the sequences that do not interact with vitamins are extracted from BioLip; then, the maximal pairwise sequence identity of the extracted protein sequences is culled to 30% by using CD-Hit [36] program, and the reduced dataset is obtained. Moreover, if a given sequence in the reduced dataset shares >30% identity with a sequence in the training dataset DVI, we remove the sequence from the reduced dataset. Finally, the remaining 6676 sequences (with 1852390 residues) constitute NVD.

All the datasets used in this study are included in Additional file 1.

### Feature representation

Feature representation is a critical step in designing a machine-learning-based predictor. In this study, multiple sequence-derived features, which potentially have a positive impact on the performance improvement of protein-vitamin binding residue prediction, are extracted and combined to form an informative feature space.

#### 1) Position-specific scoring matrix (PSSM)

Previous studies have demonstrated that the evolutionary information reflected by a position specific scoring matrix (PSSM) is a powerful feature source in many bioinformatics problems including protein-ligand binding predictions [20, 21, 37–40]. In view of this, PSSM was also taken as a feature source in this study. First, we obtained the original PSSM of a sequence by executing PSI-BLAST [41] to search the Swiss-Prot database through three iterations with 0.001 as the *E*-value cut-off against the sequence; then, each element *x* contained in PSSM was normalised by the logistic function *f*(*x*) = 1/(1 + e^− *x*^), and the normalised PSSM was obtained. Finally, the PSSM-based feature vector for each residue in the query sequence can be extracted with a sliding window as follows: for a residue at position *i* of the query sequence, its feature vector consists of the normalised PSSM elements of the query sequence corresponding to a sequence segment of length *W* centred on *i*. In this study, *W*, i.e., the size of sliding window, was set to 17, which has been demonstrated to be a better choice in VitaPred [30] and several other protein-ligand binding site prediction studies [37, 38]. Consequently, the dimensionality of the *PSSM* feature vector of a residue is 17 × 20 = 340.

#### 2) Predicted secondary structures (PSSs)

A fundamental hypothesis for most of the sequence-based protein attribute predictions is that sequences with similar structures will have similar functions. Previous studies have also shown that a close relationship exists between protein structure and function. Many structural characteristics, such as secondary structure information, have been extensively investigated for the identification of protein functional residues (e.g., protein-ligand binding residues [40, 42]). The appropriate utilisation of protein structural information may potentially help to improve the performance of protein-ligand binding prediction, as has been empirically demonstrated in our recent work [21, 38]. Therefore, protein secondary structure information, predicted from the protein sequence by performing PSIPRED [43], was used as another feature source for protein-vitamin binding residue prediction.

The predicted secondary structure information of a protein sequence is obtained by applying PSIPRED [43] software, which predicts the likelihood that a given residue in a protein sequence belongs to one of three secondary structure classes: coil (C), helix (H), and strand (E). More specifically, for a protein sequence with *L* residues, PSIPRED outputs an *L* × 3 probability matrix, which represents the predicted secondary structure information of the protein. Again, a sliding window of size 17 was used to extract the predicted secondary structure feature of each residue, and the dimensionality of the extracted *PSSs* feature vector was 17 × 3 = 51.

#### 3) Vitamin binding propensities (VBPs)

Previous studies have demonstrated that different ligands tend to bind different residues [18, 19, 21]. Panwar et al. [30] also analysed different protein-interacting residues of different vitamin classes and observed that different vitamins tend to bind different residues; this phenomenon can also be observed within vitamin sub-classes. Motivated by this observation, we can thus calculate the binding propensities of the 20 native amino acids for each type of vitamin and then extract a vitamin-specific 17-D binding propensity feature vector, denoted as *VBP*, for each residue in a protein sequence by concatenating the binding propensities of its neighbouring residues within a window of size 17 centred at the residue.

Finally, the feature representation of a residue is formed by serially combining its three corresponding feature vectors, i.e., *PSSM*, *PSSs*, and *VBPs*, and the dimensionality of the obtained feature vector is 340 + 51 + 17 = 408-D.

### Ensemble multiple heterogeneous subspace SVMs based on feature selection

After determining the feature representation, prediction models can be trained on a dataset with machine-learning algorithms such as SVM, as used in this study. However, directly training prediction models on the original feature space is often not the best solution. One important reason is that redundant information that has no positive, and sometimes even negative, impact on the prediction performance could potentially exist. Selection of the most discriminative feature subspace from the original feature space may help to improve prediction performance. Accordingly, feature selection has been a hotspot and is widely used in many bioinformatics and related fields [44], such as sequence analysis [45, 46] and microarray analysis [47, 48]. For example, our recent work [49] has demonstrated that the *PSSM* feature contains redundant information, which is useless for disulphide connectivity prediction, and the prediction performance can be further improved when the original PSSM feature is reduced to a lower but more compact feature subspace via feature selection.

However, many existing traditional feature selection methods such as data variance [49], the Fisher score [50], and the Laplacian score [51] are faced with two deficiencies: first, the importance of features is calculated individually; thus, the correlation and dependency of different feature components are neglected. Second, the dimensionality of the reduced feature subspace needs to be prescribed in advance of the feature selection process, which is often difficult or even impossible in practice.

Recently, we developed a generalised Joint Laplacian Feature Weights Learning algorithm [52], denoted as JLFWL, which can effectively address the above-mentioned two deficiencies. Rather than computing feature weights one by one, JLFWL automatically determines the optimal size of the feature subspace and selects the best feature components from the original feature space by iteratively learning the feature weights jointly and simultaneously.

Here, we briefly restate JLFWL; details can be found in [52].

Let matrix *X* = [**x**_1_, **x**_2_, ⋯, **x**_*M*_] ∈ *R*^*N* × *M*^ be a training dataset, where *M* is the number of samples, *N* is the dimensionality of features, and **x**_*i*_ is the feature vector of the *i*-th training sample. Then, the Joint Laplacian Feature Weights Learning can be summarised in Algorithm 1.


Note that in Algorithm 1, *ε* ≥ 0 is a parameter to control the  -norm of **w**. In this study, *ε* is set to be 0.5.

Different feature subspaces can be selected from the original feature space with different feature selection methods, and the discriminative characteristics of the obtained feature subspaces may also differ. Prediction models trained on these different feature subspaces potentially complement each other, which motivates us to propose an ensemble learning scheme, i.e., a multiple heterogeneous subspace SVMs ensemble based on feature selection, as follows:

First, multiple feature subspaces are selected by applying different feature selection methods; then, based on these selected feature subspaces, multiple prediction models, which are termed as heterogeneous models, can be trained on the same dataset; for a query input, the final prediction output is obtained by ensembling the outputs of the trained heterogeneous prediction models.

To demonstrate the efficacy of the proposed ensemble learning scheme, three feature selection methods (i.e., our JLFWL together with two traditional feature selection methods, such as the Fisher score [50] and Laplacian score [51]) are taken to perform feature selections, and SVM [53][54] is used as the base machine-learning algorithm to train multiple heterogeneous prediction models. We locally performed JLFWL on benchmark datasets and found that the optimal dimensionality of the feature subspace, which is automatically determined by JLFWL, is 386. For consistency, the dimensionalities of the feature subspaces obtained by Fisher score [50] and Laplacian score [51] feature selection methods are also set to be 386.

In this study, C-SVM is used and there are no specific weights for the three heterogamous SVM models. Radial basis function is chosen as the kernel function. The other two parameters, i.e., the regularisation parameter *γ* and the kernel width parameter *σ*, are set according to the optimisation results from a grid search strategy in the LIBSVM software.

### Workflow of the proposed TargetVita

**Figure 1:**
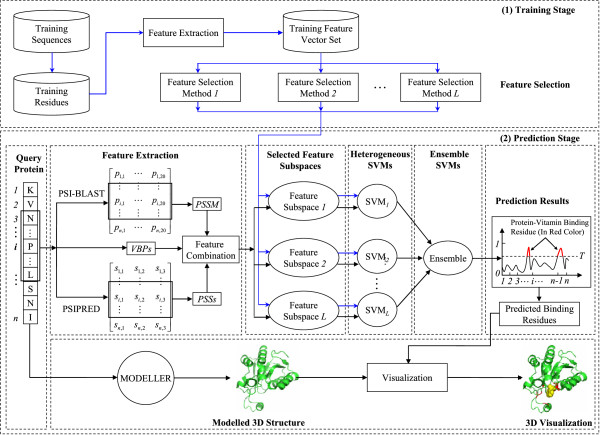
**Workflow of the proposed TargetVita.** Predicted binding residues, modelled 3D structure, and the vitamin are highlighted in red, green, and yellow colours, respectively. Arrows highlighted in blue colour denote the workflows in the training stage, while arrows in black colour denote the workflows in the prediction stage.

Figure 1 illustrates the workflow of the proposed TargetVita. In the training stage, all the feature vectors of the residues in the training sequences constitute the training feature vector set; then, *L* feature subspaces can be selected by performing *L* different feature selection methods on the training feature vector set; based on the selected feature subspace, *L* heterogamous SVM models can be trained.

In the prediction stage, for each residue in a query protein sequence, its *PSSM* and *PSSs* feature vectors are first extracted by calling PSI-BLAST and PSIPRED and applying a sliding window technique; then, its *PSSM*, *PSSs*, and *VBP* feature vectors are combined and filtered by the *L* selected feature subspaces, and the *L* filtered feature vectors (i.e., feature vectors after feature selection) are further fed to the *L* corresponding trained heterogamous SVMs that predict, for that residue, the scores related to vitamin interaction; the final score for the residue is obtained by averaging the predicted scores of those heterogeneous SVM models. Finally, a threshold *T* is used to determine whether the residue is vitamin-interacting: residues with scores above threshold are marked as vitamin-interacting.

To help with the visualisation of the prediction results, MODELLER software [55] is taken to model the protein 3D structure from the sequence, and the predicted vitamin-interacting residues are highlighted in red on the modelled 3D structure.

### Evaluation indexes

To evaluate the performance of the proposed method, four routinely used evaluation indexes in this field, i.e., *Sensitivity* (*Sn*), *Specificity* (*Sp*), *Accuracy* (*Acc*), and the Matthews correlation coefficient (*MCC*) were taken:
1234

where *TP*, *FP*, *TN*, and *FN* are the abbreviations of True Positive, False Positive, True Negative, and False Negative, respectively.

However, these four evaluation indexes are threshold-dependent, i.e., the values of these indexes vary with the threshold chosen. Clearly, it is impossible and unnecessary to report the values of these indexes under all the possible thresholds [20, 21, 37]. In light of this, two threshold selection strategies, which have been widely applied in the related fields, were taken to report the threshold-dependent evaluation indexes, i.e., *Sn*, *Sp*, *Acc*, and *MCC*.

**Strategy I:** Threshold that balances the values of *Sn* and *Sp*

The threshold-dependent evaluation indexes are reported with a threshold, denoted as *T*_*Balance*_, at which the value of *Sn* is equal or roughly equal to that of *Sp*. For convenience, the evaluation results obtained under *T*_*Balance*_ will be termed as *Balanced Evaluation* in the subsequent descriptions.

**Strategy II:** Threshold that maximises the value of *MCC*

Because the *MCC* provides the overall measurement of the quality of the binary predictions, we thus reported the threshold-dependent evaluation indexes by choosing the threshold, denoted as *T*_*MaxMCC*_, which maximises the value of *MCC* of predictions. Similarly, for convenience, the evaluation results obtained under *T*_*MaxMCC*_ will be termed as *MaxMCC Evaluation*.

In addition, to evaluate the overall prediction quality of a prediction model, the *AUC*, which is the area under the Receiver Operating Characteristic (*ROC*) curve and is threshold-independent, was also taken.

## Results and discussion

### Inappropriate cross-validation will over-estimate prediction performance

Cross-validation methods are often used to evaluate the performance of a predictor [56, 57]. Previous studies have shown that the leave-one-out cross-validation (jack-knife test) is most stringent [58–61]. However, leave-one-out cross-validation is time-consuming, especially when a dataset is huge and a complicated prediction algorithm (such as SVM in this study) is used. Additionally, because we need make a fair comparison of the proposed method with VitaPred [30], the five-fold cross-validation, which was used by Panwar et al. [30] to evaluate VitaPred, was also adopted in this study.

Another critical aspect that should be addressed here is the method of performing five-fold cross-validation. In fact, five-fold cross-validation can be performed at two different levels for the considered protein-vitamin binding residue prediction problem: (i) residue-level cross-validation and (ii) sequence-level cross-validation.

Residue-level five-fold cross-validation is performed as follows: residues in all the training protein sequences are randomly partitioned into five equally sized, disjoint subsets; then, one subset is used for testing, and the remaining four subsets are used for training; this process is continued until all the five subsets of the training dataset are traversed.

For sequence-level five-fold cross-validation, training protein sequences, rather than training residues, are randomly partitioned into five equally sized, disjoint subsets; then, residues in one subset are used for testing, and residues in the remaining four subsets are used for training; this practice is continued until all the five subsets of the training dataset are traversed.

In reference [30], Panwar et al. used residue-level cross-validation to evaluate the performance of their VitaPred. However, we believe that residue-level cross-validation tends to over-estimate the performance of a prediction model and is therefore inappropriate. Next, we will empirically demonstrate this argument as follows:

Note that to objectively and fairly compare our results with those obtained by VitaPred [30], the same machine-learning model (i.e., SVM) and the same feature representation (i.e., 340-D PSSM feature) were also used in our experiments.

For each of the four benchmark datasets (i.e., DVI, DVAI, DVBI, and DPLPI), we performed residue- and sequence-level five-fold cross-validations. Tables 2 and 3 summarise the performance comparisons between residue- and sequence-level five-fold cross-validations on the four benchmark datasets under *Balanced Evaluation* and *MaxMCC Evaluation*, respectively. Note that in Tables 2 and 3, SVM-R and SVM-S denote the results obtained by residue- and sequence-level cross-validations, respectively.

**Table 2:** **Performance comparisons between residue- and sequence-level five-fold cross-validations on DVI, DVAI, DVBI, and DPLPI under** ***Balanced Evaluation***

Dataset	Method	*Sn* (%)	*Sp* (%)	*Acc* (%)	*MCC*	*AUC*	*TP*	*TN*	*FP*	*FN*
DVI	VitaPred^*^	78.52	78.61	78.60	0.37	0.87	-	-	-	-
	SVM-R^◇^	77.88	81.34	81.18	0.30	0.87	2349	50530	11592	667
	SVM-S^△^	77.65	80.16	80.04	0.29	0.87	2342	49797	12325	674
DVAI	VitaPred^*^	72.70	76.89	76.51	0.32	0.83	-	-	-	-
	SVM-R^◇^	73.98	77.94	77.67	0.30	0.85	398	5749	1627	140
	SVM-S^△^	72.12	76.34	76.06	0.28	0.82	388	5631	1745	150
DVBI	VitaPred^*^	83.33	80.51	80.77	0.42	0.90	-	-	-	-
	SVM-R^◇^	80.44	83.83	83.68	0.33	0.90	1785	42063	8116	434
	SVM-S^△^	79.86	82.90	82.77	0.32	0.89	1772	41598	8581	447
DPLPI	VitaPred^*^	90.20	92.61	92.40	0.67	0.97	-	-	-	-
	SVM-R^◇^	91.48	93.38	93.30	0.55	0.97	999	24874	1764	93
	SVM-S^△^	90.38	92.62	92.53	0.52	0.96	987	24672	1966	105

^*^Data obtained from [30].

^◇^SVM-R: The re-implementation of VitaPred over residue-level cross-validation.

^△^SVM-S: The re-implementation of VitaPred over sequence-level cross-validation.

**Table 3:** **Performance comparisons between residue- and sequence-level five-fold cross-validations on DVI, DVAI, DVBI, and DPLPI under** ***MaxMCC Evaluation***

Dataset	Method	*Sn* (%)	*Sp* (%)	*Acc* (%)	*MCC*	*AUC*	*TP*	*TN*	*FP*	*FN*
DVI	VitaPred^*^	52.19	96.79	92.73	0.53	0.87	-	-	-	-
	SVM-R^◇^	52.62	98.29	96.18	0.54	0.87	1586	61063	1059	1430
	SVM-S^△^	52.29	98.32	96.19	0.54	0.87	1577	61076	1046	1439
DVAI	VitaPred^*^	42.75	97.51	92.54	0.48	0.83	-	-	-	-
	SVM-R^◇^	43.49	96.39	92.80	0.41	0.85	234	7110	266	304
	SVM-S^△^	40.15	96.39	92.57	0.39	0.82	216	7109	267	322
DVBI	VitaPred^*^	55.57	98.04	94.18	0.61	0.90	-	-	-	-
	SVM-R^◇^	58.77	98.45	96.77	0.59	0.90	1304	49401	778	915
	SVM-S^△^	58.18	98.40	96.69	0.58	0.89	1291	49373	806	928
DPLPI	VitaPred^*^	79.76	98.62	96.91	0.81	0.97	-	-	-	-
	SVM-R^◇^	79.67	99.19	98.42	0.79	0.97	870	26422	216	222
	SVM-S^△^	80.86	99.07	98.36	0.79	0.96	883	26391	247	209

^*^Data excerpted from [30].

^◇^SVM-R: The re-implementation of VitaPred over residue-level cross-validation.

^△^SVM-S: The re-implementation of VitaPred over sequence-level cross-validation.

In Table 2, it can be observed that the values of *Sn*, *Sp*, *Acc*, *MCC*, and *AUC* for SVM-R are consistently superior to those for SVM-S throughout the four benchmark datasets. Taking *MCC* as an example, SVM-R clearly outperforms SVM-S, and an average improvement of 2% was observed on the four benchmark datasets. Similar results can also be observed in Table 3. From the comparison results between SVM-R and SVM-S on the four considered datasets listed in Tables 2 and 3, we empirically demonstrated that the residue-level cross-validation does over-estimate the performance of a prediction model. We speculate that the main reason for this over-estimation is that during the residue-level cross-validation, some testing residues and training residues may originate from the same protein sequence and thus have much higher homology, which will lead to a better prediction performance.

On the other hand, SVM-R is in fact a re-implementation of VitaPred [30] because the prediction model and feature representation used in SVM-R are exactly the same as those used in VitaPred. By revisiting Tables 2 and 3, we can find that the *AUC* values for SVM-R on the four benchmark datasets are 0.87, 0.85, 0.90, and 0.97, which are equal to that for VitaPred with only one minor exception (i.e., 0.85 and 0.83 for SVM-R and VitaPred, respectively, in the DVAI dataset), showing that SVM-R and VitaPred achieved almost equal overall prediction quality.

However, several abnormal phenomena are observed when comparing the other four indexes, (i.e., *Sn*, *Sp*, *Acc*, and *MCC*) between SVM-R and VitaPred. Using the results in the DPLPI dataset under Balanced Evaluation as an example (refer to Table 2), the values of *Sn*, *Sp*, and *Acc* for VitaPred are 90.20%, 92.61%, and 92.40%, respectively, which are obviously lower than those for SVM-R (i.e., 91.48%, 93.38%, and 93.30%, respectively); however, the value of *MCC* for VitaPred is, unexpectedly, approximately 12% higher than that for SVM-R. A similar phenomenon can also be observed in the DVAI dataset. According to the definitions of *Sn*, *Sp*, *Acc*, and *MCC* and the relationships between them, this phenomenon should not appear. Based on the comparison between SVM-R and VitaPred, we speculate that the *MCC* reported in VitaPred [30] has been over-estimated or over-optimised, which will be further demonstrated in the subsequent independent validation test.

Considering that the residue-level cross-validation will over-estimate the performance of a prediction model, together with the fact that VitaPred may possibly have over-estimated the *MCC*s on benchmark datasets, we will take SVM-S (i.e., a re-implementation of VitaPred) over sequence-level cross-validation evaluation, rather than VitaPred itself, as the baseline predictor to demonstrate the improvements in our proposed methods in the subsequent experiments.

### Improving prediction performance by combining the features of *PSSM*, *PSSs*, and *VBPs*

**Figure 2:**
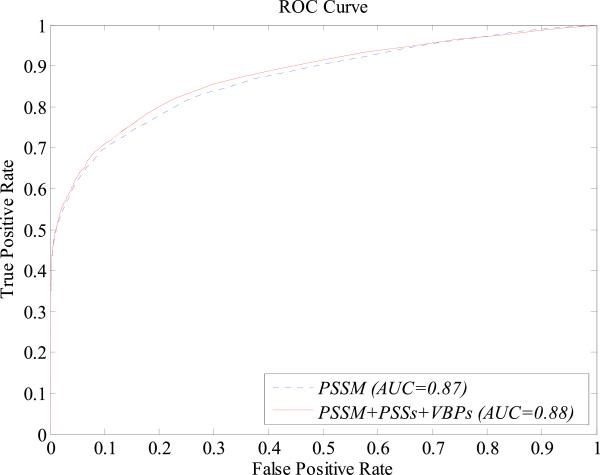
**ROC curves of the predictions with** ***PSSM*** **and** ***PSSM*** **+** ***PSSs*** **+** ***VBPs*** **features, respectively, on the DVI dataset over sequence-level five-fold cross-validation.**

In this section, we will demonstrate that the performance of protein-vitamin interaction prediction can be further improved by combining the features of *PSSM*, *PSSs*, and *VBPs*. The features of 340-D *PSSM*, 51-D *PSSs*, and 17-D *VBPs* are serially combined to form a 408-D discriminative feature, denoted as *PSSM* + *PSSs* + *VBPs*. We then evaluated the SVM-S with the *PSSM* + *PSSs* + *VBPs* feature as a model input on each of the four benchmark datasets over five-fold sequence-level cross-validation. Tables 4 and 5 summarise the performance comparisons between the *PSSM* + *PSSs + VBPs* and *PSSM* features under *Balanced Evaluation* and *MaxMCC Evaluation*, respectively. From Tables 4 and 5, we can see that the prediction performances are indeed improved on all the four benchmark datasets after incorporating *PSSs* and *VBPs* features into the *PSSM* feature under both *Balanced Evaluation* and *MaxMCC Evaluation*. Taking *MCC*, which is the overall measurement of the quality of the binary predictions, as an example, an average improvement of 1.5% was observed under both *Balanced Evaluation* and *MaxMCC Evaluation*. In terms of the *AUC*, which measures the overall prediction quality of a prediction mode, an average improvement of 1% was also observed. Figure 2 illustrates the ROC curves of the predictions with *PSSM* and *PSSM* + *PSSs* + *VBPs* features, respectively, on the DVI dataset over sequence-level five-fold cross-validation.

**Table 4:** **Performance comparisons between** ***PSSM + PSSs*** **+** ***VBPs*** **and** ***PSSM*** **features on DVI, DVAI, DVBI, and DPLPI datasets over five-fold sequence-level cross-validation under** ***Balanced Evaluation***

Dataset	Feature	*Sn* (%)	*Sp* (%)	*Acc* (%)	*MCC*	*AUC*	*TP*	*TN*	*FP*	*FN*
DVI	*PSSM*	77.65	80.16	80.04	0.29	0.87	2342	49797	12325	674
	*PSSM* + *PSSs + VBPs*	78.55	82.02	81.86	0.31	0.88	2369	50951	11171	647
DVAI	*PSSM*	72.12	76.34	76.06	0.28	0.82	388	5631	1745	150
	*PSSM* + *PSSs + VBPs*	72.12	78.28	77.86	0.29	0.84	388	5774	1602	150
DVBI	*PSSM*	79.86	82.90	82.77	0.32	0.89	1772	41598	8581	447
	*PSSM* + *PSSs + VBPs*	80.71	85.14	84.96	0.35	0.90	1791	42724	7455	428
DPLPI	*PSSM*	90.38	92.62	92.53	0.52	0.96	987	24672	1966	105
	*PSSM* + *PSSs + VBPs*	91.48	93.09	93.03	0.54	0.97	999	24798	1840	93

**Table 5:** **Performance comparisons between** ***PSSM + PSSs*** **+** ***VBPs*** **and** ***PSSM*** **on DVI, DVAI, DVBI, and DPLPI datasets over five-fold sequence-level cross-validation under** ***MaxMCC Evaluation***

Dataset	Feature	*Sn* (%)	*Sp* (%)	*Acc* (%)	*MCC*	*AUC*	*TP*	*TN*	*FP*	*FN*
DVI	*PSSM*	52.29	98.32	96.19	0.54	0.87	1577	61076	1046	1439
	*PSSM + PSSs* + *VBPs*	53.22	98.32	96.23	0.55	0.88	1605	61080	1042	1411
DVAI	*PSSM*	40.15	96.39	92.57	0.39	0.82	216	7109	267	322
	*PSSM + PSSs* + *VBPs*	44.24	96.61	93.05	0.43	0.84	238	7126	250	300
DVBI	*PSSM*	58.18	98.40	96.69	0.58	0.89	1291	49373	806	928
	*PSSM + PSSs* + *VBPs*	59.58	98.33	96.69	0.59	0.90	1322	49342	837	897
DPLPI	*PSSM*	80.86	99.07	98.36	0.79	0.96	883	26391	247	209
	*PSSM + PSSs* + *VBPs*	81.32	99.12	98.42	0.79	0.97	888	26403	235	204

We have also provided performance comparisons of different feature combinations in Additional file 2: Table S1.

### Ensembling heterogeneous SVMs helps to improve the prediction performance

**Figure 3:**
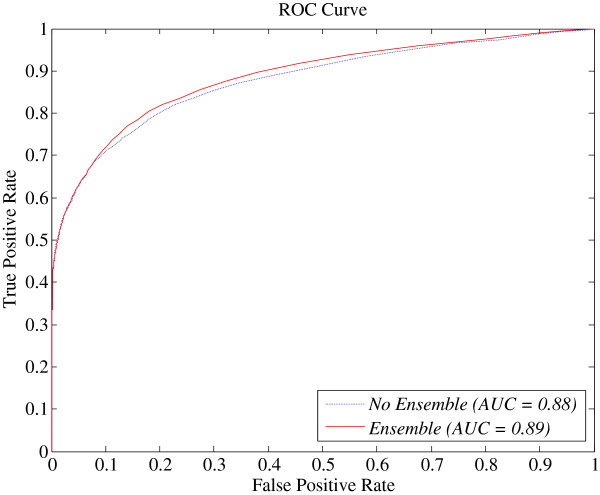
**ROC curves of the predictions with ensemble and no ensemble, respectively, on the DVI dataset over sequence-level five-fold cross-validation.**

In this section, we will empirically demonstrate that the prediction performance of protein-vitamin interactions can be further improved by ensembling multiple heterogeneous SVMs. More specifically, we adopted three feature selection methods (i.e., data variance [49], Fisher score [50], and Laplacian score [51]) to select three different feature subsets from the original *PSSM* + *PSSs* + *VBPs* feature space; then, we trained three heterogeneous SVMs on the selected feature subsets. The final prediction was performed by averaging the outputs of the three trained SVMs. For comparison, we also directly trained an SVM with the original *PSSM* + *PSSs* + *VBPs* feature, denoted as no ensemble. Table 6 summarises the performance comparisons between ensemble and no ensemble on all the four considered datasets over sequence-level five-fold cross-validation under *Balanced Evaluation*. Figure 3 illustrates the ROC curves of the predictions with ensemble and no ensemble, respectively, on the DVI dataset over sequence-level five-fold cross-validation.

**Table 6:** **Performance comparisons between with- and without-ensemble on DVI, DVAI, DVBI, and DPLPI datasets over five-fold sequence-level cross-validation under** ***Balanced Evaluation***

Dataset	Ensemble	*Sn* (%)	*Sp* (%)	*Acc* (%)	*MCC*	*AUC*	*TP*	*TN*	*FP*	*FN*
DVI	No	78.55	82.02	81.86	0.31	0.88	2369	50951	11171	647
	Yes	78.45	84.17	83.90	0.34	0.89	2366	52285	9837	650
DVAI	No	72.12	78.28	77.86	0.29	0.84	388	5774	1602	150
	Yes	72.68	79.89	79.40	0.31	0.85	391	5893	1483	147
DVBI	No	80.71	85.14	84.96	0.35	0.90	1791	42724	7455	428
	Yes	81.34	85.49	85.31	0.36	0.91	1805	42898	7281	414
DPLPI	No	91.48	93.09	93.03	0.54	0.97	999	24798	1840	93
	Yes	91.30	93.65	93.56	0.56	0.97	997	24947	1691	95

In Table 6, it can be observed that the values of the five evaluation indexes (i.e., *Sn*, *Sp*, *Acc*, *MCC*, and *AUC*) of the prediction under ensemble are consistently superior to that of the prediction under no ensemble, with only two exceptions: *Sn* in the DVI and DPLPI datasets. The prediction under ensemble achieved average improvements of approximately 2% and 1% for *MCC* and *AUC*, respectively. The results demonstrate that the heterogeneous SVMs trained with different feature subsets can complement with each other, which accounts for the improvements in the prediction performance.

### Comparison with existing protein-vitamin interaction predictors

In this section, we will compare the proposed method, called TargetVita, with existing predictors for protein-vitamin prediction. Note that in TargetVita, the *PSSM* + *PSSs + VBPs* feature was used as the input feature, and the SVM ensemble based on feature selection was applied. To the best of our knowledge, VitaPred [30] is the only predictor that was specifically designed for protein-vitamin prediction; thus, we compare the proposed TargetVita with VitaPred under both a cross-validation test and an independent validation test.

However, the cross-validation performance of VitaPred may have been overestimated because it was evaluated by performing residue-level cross-validation. In view of this, SVM-S, which is a re-implementation of VitaPred that was evaluated over sequence-level cross-validation, was taken to compare with the proposed TargetVita when performing the cross-validation test. As for the independent validation test, TargetVita will be compared with both VitaPred and the SVM-S.

#### A. Cross-validation test

Tables 7 and 8 illustrate the performance comparisons between TargetVita and SVM-S on the four datasets over five-fold sequence-level cross-validation under *Balanced Evaluation* and *MaxMCC Evaluation*, respectively. From Table 7, we can see that the values of the five evaluation indexes for TargetVita are consistently superior to that of SVM-S throughout the four benchmark datasets. Taking *MCC* and *AUC* as examples, which are the two indexes measuring the overall prediction performance of a predictor, TargetVita clearly outperforms SVM-S, and average improvements of 3.5% and 2% were observed, respectively. Under *MaxMCC Evaluation* (refer to Table 8), a similar phenomenon can also be observed, with only minor exceptions on *Sn*.

**Table 7:** **Performance comparisons with SVM-S on DVI, DVAI, DVBI, and DPLPI datasets over five-fold sequence-level cross-validation under** ***Balanced Evaluation***

Dataset	Method	*Sn* (%)	*Sp* (%)	*Acc* (%)	*MCC*	*AUC*	*TP*	*TN*	*FP*	*FN*
DVI	SVM-S^△^	77.65	80.16	80.04	0.29	0.87	2342	49797	12325	674
	TargetVita	78.45	84.17	83.90	0.34	0.89	2366	52285	9837	650
DVAI	SVM-S^△^	72.12	76.34	76.06	0.28	0.82	388	5631	1745	150
	TargetVita	72.68	79.89	79.40	0.31	0.85	391	5893	1483	147
DVBI	SVM-S^△^	79.86	82.90	82.77	0.32	0.89	1772	41598	8581	447
	TargetVita	81.34	85.49	85.31	0.36	0.91	1805	42898	7281	414
DPLPI	SVM-S^△^	90.38	92.62	92.53	0.52	0.96	987	24672	1966	105
	TargetVita	91.30	93.65	93.56	0.56	0.97	997	24947	1691	95

^△^SVM-S: The re-implementation of VitaPred over sequence-level cross-validation.

**Table 8:** **Performance comparisons with existing predictors on DVI, DVAI, DVBI, and DPLPI datasets over five-fold sequence-level cross-validation under** ***MaxMCC Evaluation***

Dataset	Method	*Sn* (%)	*Sp* (%)	*Acc* (%)	*MCC*	*AUC*	*TP*	*TN*	*FP*	*FN*
DVI	SVM-S^△^	52.29	98.32	96.19	0.54	0.87	1577	61076	1046	1439
	TargetVita	51.06	98.59	96.39	0.55	0.89	1540	61244	878	1476
DVAI	SVM-S^△^	40.15	96.39	92.57	0.39	0.82	216	7109	267	322
	TargetVita	44.43	96.81	93.25	0.44	0.85	239	7141	235	299
DVBI	SVM-S^△^	58.18	98.40	96.69	0.58	0.89	1291	49373	806	928
	TargetVita	56.21	98.81	97.02	0.60	0.91	1248	49582	597	971
DPLPI	SVM-S^△^	80.86	99.07	98.36	0.79	0.96	883	26391	247	209
	TargetVita	74.05	99.61	98.60	0.80	0.97	812	26534	104	280

^△^SVM-S: The re-implementation of VitaPred over sequence-level cross-validation.

#### B. Independent validation test

Performing only cross-validation comparisons to demonstrate the effectiveness of a newly developed method over an existing method is often not convincing, the reason being that the characteristics of the new method may be *over-fitted* and/or *over-optimised* to the underlying dataset for the purpose of pursuing positive comparison results [21, 62, 63]. Validation on fresh independent data has been considered as an important and necessary procedure when comparing different methods, and it has been widely applied in related research.

With this view, we also performed independent validation tests to further demonstrate the superiority of the proposed TargetVita over existing protein-vitamin predictors. Tables 9 and 10 summarise the performance comparisons between TargetVita and existing predictors on independent validation tests under *Balanced Evaluation* and *MaxMCC Evaluation*, respectively. From Tables 9 and 10, two observations can be made as follows:

**Table 9:** **Performance comparisons with existing predictors on the independent validation datasets under** ***Balanced Evaluation***

Dataset	Method	*Sn* (%)	*Sp* (%)	*Acc* (%)	*MCC*	*AUC*	*TP*	*TN*	*FP*	*FN*
DVI	VitaPred^*^	73.70	71.98	72.07	0.22	-	**-**	**-**	**-**	**-**
	SVM-S^△^	75.38	78.51	78.35	0.28	0.85	493	9167	2509	161
	TargetVita	80.73	81.05	81.03	0.33	0.89	528	9463	2213	126
DVAI	VitaPred^*^	73.48	72.87	72.93	0.31	-	-	-	-	-
	SVM-S^△^	73.48	79.25	78.61	0.38	0.83	133	1142	299	48
	TargetVita	79.01	79.18	79.16	0.41	0.86	143	1141	300	38
DVBI	VitaPred^*^	83.05	68.76	69.40	0.23	-	-	-	-	-
	SVM-S^△^	78.28	81.49	81.35	0.30	0.88	328	7291	1656	91
	TargetVita	81.38	81.69	81.68	0.32	0.90	341	7309	1638	78
DPLPI	VitaPred^*^	84.15	83.22	83.26	0.33	-	-	-	-	-
	SVM-S^△^	85.77	90.18	90.00	0.44	0.95	211	5352	583	35
	TargetVita	89.02	89.30	89.29	0.44	0.96	219	5300	635	27

^*^Data excepted from [30].

^△^SVM-S: The re-implementation of VitaPred.

**Table 10:** **Performance comparisons with existing predictors on the independent validation datasets under** ***MaxMCC Evaluation***

Dataset	Method	*Sn* (%)	*Sp* (%)	*Acc* (%)	*MCC*	*AUC*	*TP*	*TN*	*FP*	*FN*
DVI	VitaPred^*^	41.74	96.63	93.72	0.38	-	-	-	-	-
	SVM-S^△^	47.09	98.40	95.68	0.52	0.85	308	11489	187	346
	TargetVita	47.01	98.42	95.69	0.52	0.89	308	11491	185	346
DVAI	VitaPred^*^	30.39	97.22	89.77	0.37	-	-	-	-	-
	SVM-S^△^	32.04	97.09	89.83	0.38	0.83	58	1399	42	123
	TargetVita	38.12	96.81	90.26	0.43	0.86	69	1395	46	112
DVBI	VitaPred^*^	49.40	94.49	92.47	0.35	-	-	-	-	-
	SVM-S^△^	52.03	98.25	96.18	0.53	0.88	218	8790	157	201
	TargetVita	51.06	98.69	96.56	0.55	0.90	214	8830	117	205
DPLPI	VitaPred^*^	65.85	98.40	97.10	0.63	-	-	-	-	-
	SVM-S^△^	72.76	99.11	98.06	0.74	0.95	179	5882	53	67
	TargetVita	74.39	99.07	98.09	0.75	0.96	183	5880	55	63

^*^Data excepted from [30].

^△^SVM-S: The re-implementation of VitaPred.

First, the proposed TargetVita significantly outperforms VitaPred under both *Balanced Evaluation* and *MaxMCC Evaluation*. Taking *MCC* as an example, TargetVita achieved approximately 9% ~ 11% and 6% ~ 20% improvements on the four independent validation datasets under *Balanced Evaluation* and *MaxMCC Evaluation*, respectively. In addition, TargetVita also outperformed SVM-S, which is a re-implementation of VitaPred, and acted as the best performer with an average improvement of approximately 2.5% for *MCC* if compared with the second best performer, SVM-S.

Second, the values of *MCC* for VitaPred over the cross-validation test and independent validation test differ significantly under both the *Balanced Evaluation* and *MaxMCC Evaluation* if compared with TargetVita and SVM-S. In other words, the performance evaluated over the cross-validation test is significantly better than that evaluated over the independent validation test for VitaPred, while similar performances were obtained over the cross-validation test and the independent validation test for TargetVita and SVM-S.

**Figure 4:**
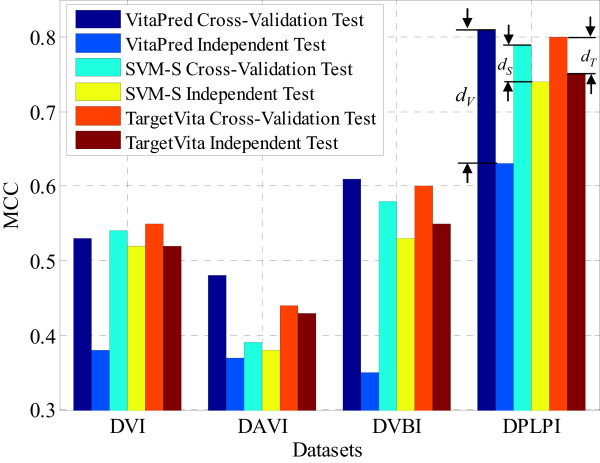
**Differences between the** ***MCC*** **values over the cross-validation test and over the independent validation test for VitaPred, SVM-S, and TargetVita on the four considered vitamins under** ***MaxMCC Evaluation*** **.**

Taking the results under *MaxMCC Evaluation* as an example, the values of *MCC* over the cross-validation test for VitaPred are 0.53, 0.48, 0.61, and 0.81, respectively (refer to Table 3), while the values of *MCC* over the independent validation test for VitaPred are 0.38, 0.37, 0.35, and 0.63, respectively (refer to Table 10), for the four considered vitamins. Then, we can calculate that the *MCC* differences between the cross-validation test and the independent validation test of VitaPred for the four considered vitamins are 0.15, 0.11, 0.26, and 0.18, respectively. By revisiting Table 8, together with Table 10, we calculate that the *MCC* differences between the cross-validation test and the independent validation test of the proposed TargetVita for the four considered vitamins are only 0.03, 0.01, 0.05, and 0.05, respectively. Similarly, the *MCC* differences of SVM-S can also be calculated from Table 3 and Table 10. Figure 4 illustrates the differences between the *MCC* values over the cross-validation test and the independent validation test for VitaPred, SVM-S, and TargetVita on the four considered vitamins under *MaxMCC Evaluation*. Note that in Figure 4, *d*_*V*_, *d*_*S*_, and *d*_*T*_ denote the *MCC* differences for VitaPred, SVM-S, and TargetVita, respectively, and only the *MCC* differences on the DPLPI dataset are explicitly labelled.

From Figure 4, we can intuitively find that the proposed TargetVita and SVM-S achieve similar performances (in terms of *MCC*) over both the cross-validation test and independent validation test, as their *MCC* differences are small, while the performance of VitaPred over the independent validation test is significantly lower than that that over the cross-validation test with all four considered vitamins, indicating that the performance of VitaPred over the cross-validation test has potentially been over-estimated or over-optimised, thus leading to a lower generalisation capability (i.e., poor performance with independent fresh data). This observation further supports the speculation (i.e., the *MCC* of VitaPred has potentially been over-estimated) we made in a previous section.

#### C. Performance on a non-vitamin binding dataset

We then performed performance comparisons between the proposed TargetVita and VitaPred on the non-vitamin binding dataset NVD, and the results of the comparison are listed in Additional file 2: Table S2. Note that the results of VitaPred and TargetVita were obtained by feeding the 6676 sequences to their corresponding web servers with default threshold settings.

From Table S2, we can clearly see that the proposed TargetVita achieved much better prediction performance than VitaPred. Among the 1852390 residues in the 6676 non-vitamin binding sequences, 46319 residues were mistakenly predicted as binding residues by VitaPred, while only 36361 false positives were obtained by TargetVita.

## Conclusions

In this study, we have designed and implemented a new sequence-based predictor, called TargetVita, for protein-vitamin binding residue prediction. TargetVita performs prediction by utilising multiple features derived from protein sequences and effectively ensembling heterogeneous SVMs trained on different feature subspaces. Experimental results on benchmark datasets demonstrated that the proposed TargetVita can achieve good performance and is superior to existing protein-vitamin binding residue predictors.

Our future work will focus on further improving the prediction performance of TargetVita by uncovering new effective feature sources and applying more powerful machine-learning algorithms.

### Availability of supporting data

The data sets supporting the results of this article are included within the Additional file 1.

## Electronic supplementary material

Additional file 1: **Datasets used in this study.** (PDF 191 KB)

Additional file 2: **Table S1.** Performance comparisons of different feature combinations over 5-fold sequence-level cross-validation under *MaxMCC Evaluation*. **Table S2.** Performance comparisons between the proposed TargetVita and VitaPred on the non-vitamin binding dataset NVD. (PDF 165 KB)
