# Supplementary material for: Enhancing protein-vitamin binding residues prediction by multiple heterogeneous subspace SVMs ensemble
Source: BMC Bioinformatics. 2014 Sep 5;15(1):297. doi: 10.1186/1471-2105-15-297 (PMC4261549; doi:10.1186/1471-2105-15-297)
Supplement: Supplementary file 1 — Additional file 1: Datasets used in this study. (PDF 191 KB) [file 12859_2014_6690_MOESM1_ESM.pdf]

Additional File 1: Datasets Used in This Study

Enhancing Protein-Vitamin Binding Residues Prediction by Multiple Heterogeneous Subspace SVMs Ensemble

Dong-Jun Yu[1,*], Jun Hu[1], Hui Yan[1], Xi-Bei Yang[1], and Jing-Yu Yang[1]

[1] School of Computer Science and Engineering, Nanjing University of Science and Technology, Xiaolingwei 200, Nanjing, China, 210094

[2] Institute of Image Processing and Pattern Recognition, Shanghai Jiao Tong University, Dongchuan Road 800, Shanghai, China, 200240

* Address correspondence to D.J. Yu njyudj@njust.edu.cn or H.B. Shen at hbshen@sjtu.edu.cn

Tel: +86-21-34205320

Fax: +86-21-34204022

CONTENTS

READ ME....2

DVI Training Dataset....3

DVI Independent Validation Dataset....54

DVAI Training Dataset....65

DVAI Independent Validation Dataset....71

DVBI Training Dataset....73

DVBI Independent Validation Dataset....115

DPLPI Training Dataset....122

DPLPI Independent Validation Dataset....144

Non-vitamin Binding Dataset (NVD)....149
