# Supplementary material for: Enhancing protein-vitamin binding residues prediction by multiple heterogeneous subspace SVMs ensemble
Source: BMC Bioinformatics. 2014 Sep 5;15(1):297. doi: 10.1186/1471-2105-15-297 (PMC4261549; doi:10.1186/1471-2105-15-297)
Supplement: Supplementary file 2 — Additional file 2: Table S1. Performance comparisons of different feature combinations over 5-fold sequence-level cross-validation under MaxMCC Evaluation. Table S2. Performance comparisons between the proposed TargetVita and VitaPred on the non-vitamin binding dataset NVD. (PDF 165 KB) [file 12859_2014_6690_MOESM2_ESM.pdf]

Additional file 2: Table S1-S2

Enhancing Protein-Vitamin Binding Residues Prediction by Multiple Heterogeneous Subspace SVMs Ensemble

Dong-Jun Yu[1,*], Jun Hu[1], Hui Yan[1], Xi-Bei Yang[1], and Jing-Yu Yang[1]

[1] School of Computer Science and Engineering, Nanjing University of Science and Technology, Xiaolingwei 200, Nanjing, China, 210094

[2] Institute of Image Processing and Pattern Recognition, Shanghai Jiao Tong University, Dongchuan Road 800, Shanghai, China, 200240

* Address correspondence to D.J. Yu njyudj@njust.edu.cn or H.B. Shen at hbshen@sjtu.edu.cn

Tel: +86-21-34205320

Fax: +86-21-34204022
